# The splicing factor Prpf31 is required for hematopoietic stem and progenitor cell expansion during zebrafish embryogenesis

**DOI:** 10.1016/j.jbc.2024.105772

**Published:** 2024-02-19

**Authors:** Yuexia Lv, Jingzhen Li, Shanshan Yu, Yangjun Zhang, Hualei Hu, Kui Sun, Danna Jia, Yunqiao Han, Jiayi Tu, Yuwen Huang, Xiliang Liu, Xianghan Zhang, Pan Gao, Xiang Chen, Mark Thomas Shaw Williams, Zhaohui Tang, Xinhua Shu, Mugen Liu, Xiang Ren

**Affiliations:** 1 Key Laboratory of Molecular Biophysics of the Ministry of Education, College of Life Science and Technology, Huazhong University of Science and Technology, Wuhan, China; 2 Department of Prenatal Diagnosis Center, The Third Affiliated Hospital of Zhengzhou University, Zhengzhou, China; 3 Research Center for Biochemistry and Molecular Biology, Jiangsu Key Laboratory of Brain Disease Bioinformation, Xuzhou Medical University, Xuzhou, China; 4 Institute of Visual Neuroscience and Stem Cell Engineering, College of Life Sciences and Health, Wuhan University of Science and Technology, Wuhan, China; 5 Department of Urology, Tongji Hospital, Tongji Medical College, Huazhong University of Science and Technology, Wuhan, China; 6 Department of Biological and Biomedical Sciences, Glasgow Caledonian University, Glasgow, United Kingdom

**Keywords:** pre-mRNA processing factor 31 (Prpf31), RNA alternative splicing, hematopoietic stem and progenitor cells (HSPCs), expansion, mitotic malformation, zebrafish

## Abstract

Pre-mRNA splicing is a precise regulated process and is crucial for system development and homeostasis maintenance. Mutations in spliceosomal components have been found in various hematopoietic malignancies (HMs) and have been considered as oncogenic derivers of HMs. However, the role of spliceosomal components in normal and malignant hematopoiesis remains largely unknown. Pre-mRNA processing factor 31 (PRPF31) is a constitutive spliceosomal component, which mutations are associated with autosomal dominant retinitis pigmentosa. *PRPF31* was found to be mutated in several HMs, but the function of PRPF31 in normal hematopoiesis has not been explored. In our previous study, we generated a *prpf31* knockout (KO) zebrafish line and reported that Prpf31 regulates the survival and differentiation of retinal progenitor cells by modulating the alternative splicing of genes involved in mitosis and DNA repair. In this study, by using the *prpf31* KO zebrafish line, we discovered that *prpf31* KO zebrafish exhibited severe defects in hematopoietic stem and progenitor cell (HSPC) expansion and its sequentially differentiated lineages. Immunofluorescence results showed that Prpf31-deficient HSPCs underwent malformed mitosis and M phase arrest during HSPC expansion. Transcriptome analysis and experimental validations revealed that Prpf31 deficiency extensively perturbed the alternative splicing of mitosis-related genes. Collectively, our findings elucidate a previously undescribed role for Prpf31 in HSPC expansion, through regulating the alternative splicing of mitosis-related genes.

The spliceosome is a highly dynamic and complex cellular machine, which catalyzes the splicing of pre-mRNAs, by removing introns and ligating exons to produce mature mRNAs (1). Most eukaryotic genes contain multiple exons and introns, and approximately 95% of these multiexon genes undergo alternative splicing (AS) (2). Aberrant pre-mRNA splicing is associated with several human diseases in which it can contribute toward disease severity (3, 4). The spliceosome is composed of five uridine-rich snRNAs (U1-, U2-, U4-, U5-, and U6-snRNA) and numerous associated proteins. Recently, several spliceosomal components have been found to be mutated in various hematopoietic malignancies (HMs), including *SF3B1*, *U2AF1*, *SRSF2*, *ZRSR2*, *SF3A1*, *U2AF2*, *PRPF8*, *PRPF40B*, *SF1*, *SNRNP200*, *LUC7L2*, etc (5, 6). Compared with the researches of spliceosomal component in HMs, the functions of spliceosomal components in hematopoietic development are largely unknown.

The pre-mRNA processing factors (PRPFs), which include PRPF3, PRPF4, PRPF6, PRPF8, PRPF31, PAP-1, and SNRNP200, are constitutive spliceosomal components. PRPFs are involved in the assembly of the U4/U6·U5 tri-snRNPs. Mutations in PRPFs represent the second most frequent cause of autosomal dominant retinitis pigmentosa (adRP) (7, 8, 9). PRPF31, a core spliceosomal component, is a part of the U4 snRNP (10). Mutations in *PRPF31* caused defects in RNA splicing. Lymphoblasts, derived from *PRPF31* adRP patients, showed a nearly 40% decrease in the quantitative assembly of tri-snRNPs (11). Intriguingly, two PRPFs, PRPF8 and SNRNP200，were reported to be mutated in patients with HMs (12). As PRPF31 can interact with PRPF8 and SNRNP200 (13), it is possible that PRPF31 plays a role in HMs. Notably, the COSMIC database shows 16 somatic mutations of *PRPF31* gene in a total of 7278 hematopoietic and lymphoid primary tissue samples (https://cancer.sanger.ac.uk/cosmic). This implies the potential involvement of PRPF31 in HMs.

In vertebrates, hematopoiesis consists of two successive and dynamic waves, designated as primitive hematopoiesis and definitive hematopoiesis. Primitive hematopoiesis produces embryonic erythrocytes to transport oxygen and primitive myeloid cells for immunoprotection in early embryogenesis. The definitive hematopoietic wave gives rise to hematopoietic stem and progenitor cells (HSPCs), which possess the capabilities of self-renewal and differentiation into all lineages of mature blood cells throughout life (14). Zebrafish has emerged as an excellent animal model to study vertebrate hematopoiesis and HMs (15). In zebrafish, HSPCs emerge directly from the ventral wall of the dorsal aorta in the aorta-gonad-mesonephros (AGM) region, through a process termed the endothelial-to-hematopoietic transition, at 26 h postfertilization (hpf) (16, 17). Then, the nascent HSPCs migrate to and reside in the caudal hematopoietic tissue (CHT), for transient expansion upon 36 hpf. Finally, they will colonize to the definitive hematopoietic organs, the thymus at 3 days postfertilization (dpf) and kidney at 4 dpf, for life-long hematopoiesis (16, 18, 19, 20). Several studies have demonstrated the involvement of spliceosomal components in zebrafish hematopoietic development (21, 22, 23, 24, 25, 26, 27). The spatiotemporal transcriptome analysis of the zebrafish CHT region during HSPC expansion indicated overt expression of *prpf31* in a series of hematopoietic cells and regions, including endothelial cells, HSPCs, hemogenic endothelium, caudal artery, caudal vein, and caudal vein plexus (28). This indicates the potential involvement of Prpf31 in zebrafish hematopoiesis.

In our preliminary studies, we constructed a *prpf31* knockout zebrafish model using CRISPR/Cas9 technology. We demonstrated that Prpf31 deficiency compromised the mitosis and differentiation of retinal progenitor cells, by interfering with the AS and expression of genes involved in spindle organization and DNA repair (29). Here, we clarify, for the first time that Prpf31 is required for HSPC expansion during zebrafish embryogenesis. Prpf31 deficiency disrupts the AS of mitosis-related genes and triggers malformed mitosis within HSPCs. Consequently, the expansion and differentiation of HSPCs are impaired. Our findings provide novel insights into the role of Prpf31 and a clear rationale for undertaking further functional investigations into the role of Prpf31 in normal hematopoiesis and HMs.

## Results

### *prpf31* is expressed in domains of hematopoiesis in zebrafish

**Figure 1:**
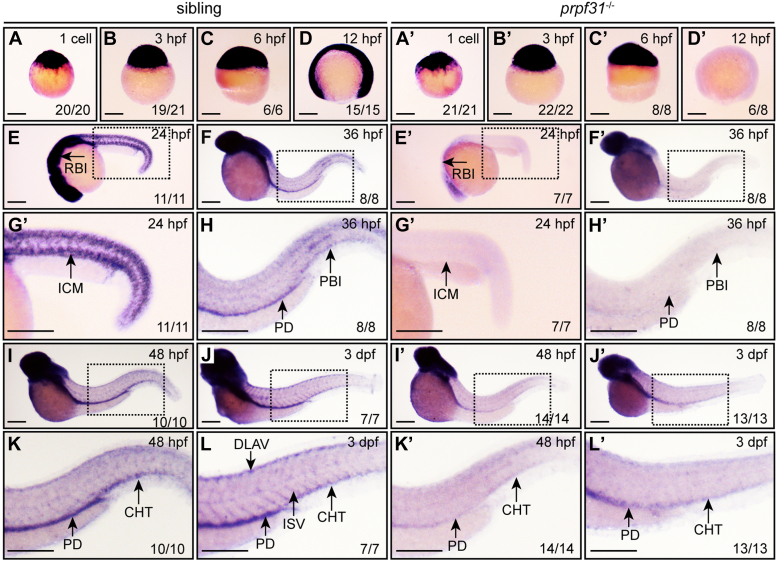
**Expression patterns of *prpf31* during zebrafish embryonic development in siblings and *prpf31***^**−/−**^**zebrafish.***A*–*L* and *A’*–*L’*, *prpf31* expression in siblings and *prpf31*^−/−^ zebrafish was examined by WISH at different developmental stages. *E*–*L* and *E’*–*L’*: lateral views, anterior to the *left* and dorsal upward. *G* and *G’*: magnified views of the ICM in (*E* and *E’*). *H* and *H’*: magnified views of the PBI in (*F* and *F’*). *K*, *L*, *K’*, and *L’*: magnified views of the CHT in (*I*, *J*, *I’*, and *J*’). The number of embryos with similar gene expression patterns among all embryos examined was shown at the *bottom right* of each panel. The scale bars represent 250 μm. CHT, caudal hematopoietic tissue; HSPC, hematopoietic stem and progenitor cell; ICM, intermediate cell mass; PBI, posterior blood island; PRPF, pre-mRNA processing factor; WISH, whole-mount *in situ* hybridization.

In our preliminary studies, we have established a homozygous *prpf31* knockout zebrafish line (hereafter referred to as *prpf31*^−/−^) using CRISPR/Cas9 technology, which was predicted to produce a truncated Prpf31 protein (p.S55fs∗102) (29). The remaining *prpf31* mRNA and functional Prpf31 protein were significantly reduced in primitive and definitive hematopoietic sites of *prpf31*^−/−^ embryos from 24 hpf onward (Fig. S1). To determine the potential function of Prpf31 in embryonic hematopoietic development, we examined the spatiotemporal expression pattern of *prpf31* by using whole-mount *in situ* hybridization (WISH), in both wild-type (WT) siblings (*prpf31*^+/+^ embryos bred from male and female *prpf31*^+/−^ parents) and *prpf31*^−/−^ zebrafish (*prpf31*^−/−^ embryos bred from male and female *prpf31*^+/−^ parents). Zebrafish *prpf31* was maternally and ubiquitously expressed until 6 hpf in both WT siblings and *prpf31*^−/−^ embryos (Fig. 1, *A*–*C* and *A’*–*C’*). At 12 hpf, *prpf31* was still ubiquitously expressed in siblings (Fig. 1*D*), whereas almost undetectable in *prpf31*^−/−^ zebrafish (Fig. 1*D’*). In WT siblings, *prpf31* was predominantly expressed in the head region, spinal cord, rostral blood island (RBI), and intermediate cell mass (ICM) at 24 hpf (Fig. 1, *E* and *G*). From 36 hpf onward, *prpf31* was diffusely expressed in the head region and pronephric duct (Fig. 1, *F*, *H*, *I*, *J*, *K*, and *L*). Additionally, expression of *prpf31* was also detected in sites associated with active hematopoiesis and vasculogenesis, namely the posterior blood island at 36 hpf (Fig. 1, *F* and *H*); CHT at 48 hpf (Fig. 1, *I* and *K*); and CHT, intersegmental blood vessels, and dorsal longitudinal anastomotic vessels at 72 hpf (Fig. 1, *J* and *L*). However, in *prpf31*^−/−^ zebrafish, from 24 hpf onward, expression of *prpf31* in the aforementioned hematopoietic and vasculature regions was almost absent (Fig. 1, *E’*–*L’*). The spatiotemporal expression profile of *prpf31* suggests that zebrafish *prpf31* might be involved in the development of hematopoiesis and vasculogenesis.

### Primitive hematopoiesis and EMP formation are intact in *prpf31*^−/−^ zebrafish

In zebrafish, RBI and ICM give rise to primitive myeloid and primitive erythroid progenitor cells, respectively. Posterior blood island represents the site of multilineage erythromyeloid-progenitor (EMP) formation (15). To determine whether primitive hematopoiesis and EMP formation develop normally within *prpf31*^−/−^ zebrafish, we conducted WISH to examine the expression of a series of primitive hematopoietic cell and EMP markers, in both WT siblings and *prpf31*^−/−^ zebrafish at 24 and 36 hpf, respectively. The hemangioblast marker *scl* and *fli1* (Fig. S2, *A*, *A’*, *B* and *B’*), myeloid progenitor marker *spi1b* (Figs. S2, *C* and *C’* and S3, *A* and *A’*), granulocyte cell marker *mpx* (Figs. S2, *E* and *E’* and S3, *B* and *B’*), neutrophils marker *lyz* (Figs. S2, *G* and *G’* and S3, *C* and *C’*), monocytes/macrophages marker *l-plastin* (Figs. S2, *I* and *I’* and S3, *D* and *D’*), erythrocyte progenitor marker *gata1a* (Figs. S2, *D* and *D’* and S3, *F* and *F’*), and the erythrocyte marker *hbae1.1, hbae3,* and *hbbe1* (Figs. S2, *F*, *F’*, *H*, *H’*, *J* and *J’* and S3, *G*, *G’*, *H*, *H’*, *I* and *I’*) were expressed at similar levels in siblings and *prpf31*^−/−^ zebrafish at 24 and 36 hpf. O-Dianisidine staining of erythrocytes and Sudan black B staining of neutrophils at 36 hpf also showed similar staining signal in siblings and *prpf31*^−/−^ zebrafish (Fig. S3, *E*, *E’*, *J* and *J’*). Moreover, live imaging of *prpf31*^−/−^ zebrafish showed identical blood circulation with sibling zebrafish at 36 hpf (Video S1 and S2). These results suggest that primitive hematopoiesis (Fig. S2) and EMP formation (Fig. S3) are intact in *prpf31*^−/−^ zebrafish.

### Definitive hematopoiesis is impaired in *prpf31*^−/−^ zebrafish

**Figure 2:**
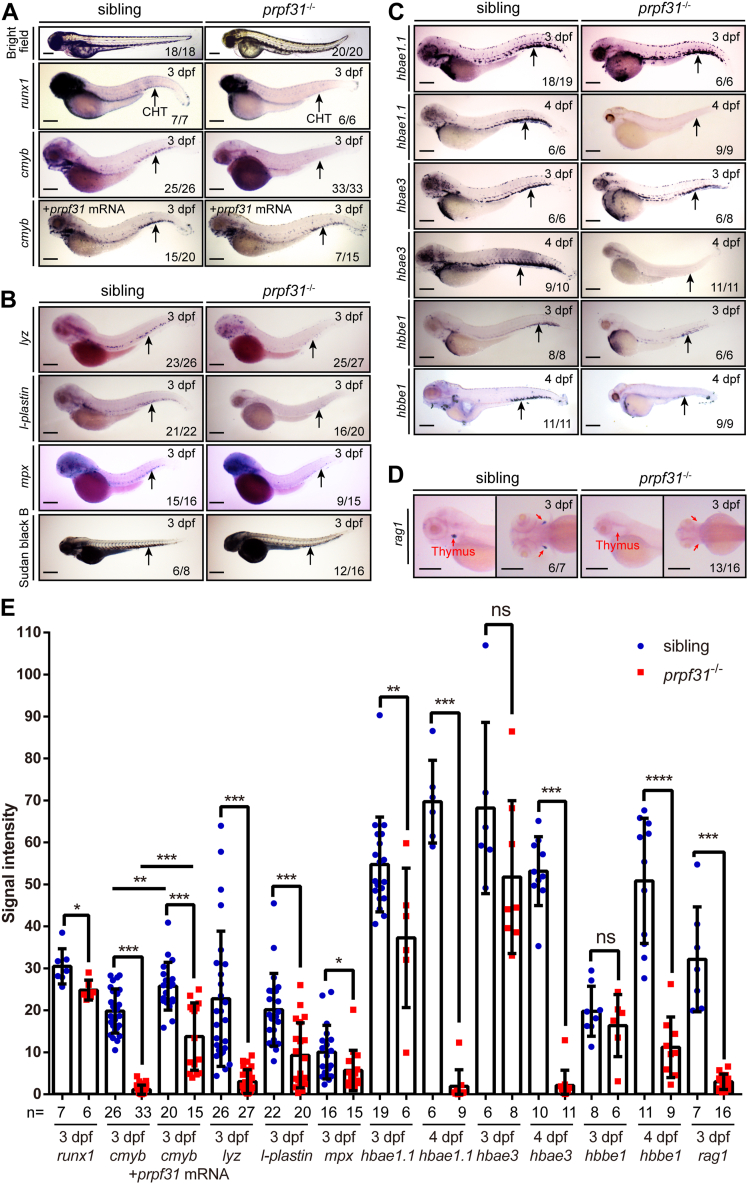
**Definitive hematopoiesis is impaired in *prpf31***^**−/−**^**zebrafish.***A*, bright field observation showed morphological abnormalities in *prpf31*^−/−^ zebrafish at 3 dpf. WISH manifested the expression of HSPC markers *runx1* and *cmyb* were noticeably reduced and rescue of *cmyb* expression after *prpf31* mRNA injection. Lateral views. *B*, expression of the myelocyte markers *lyz*, *l-plastin*, and *mpx* by WISH, and staining signal of Sudan black B labeled neutrophils were significantly decreased in the CHT of *prpf31*^−/−^ zebrafish at 3 dpf. Lateral views. *C*, expression of the erythrocyte markers *hbae1.1*, *hbae3*, and *hbbe1* were almost undetectable in the CHT of *prpf31*^−/−^ zebrafish at 4 dpf, although there was only slightly decrease compared with siblings at 3 dpf by WISH. Lateral views. *D*, expression of the early T cell marker *rag1* was almost completely absent in the thymus of *prpf31*^−/−^ zebrafish at 3 dpf by WISH. Lateral and dorsal views. *Black arrows* denote the CHT region. *Red arrows* denote the thymus region. The number of embryos with similar gene expression patterns among all embryos examined was shown at the *bottom right* of each panel. The scale bars represent 250 μm. *E*, quantification of mRNA signals of *prpf31*^−/−^ zebrafish and siblings detected by WISH in (*A*–*D*). The total number of embryos examined was indicated below each column. Mean ± SD; unpaired two-tailed *t* test; ∗*p* < 0.05, ∗∗*p* < 0.01, ∗∗∗*p* < 0.001; ns, not significant. CHT, caudal hematopoietic tissue; dpf, days postfertilization; hpf, hours postfertilization; HSPC, hematopoietic stem and progenitor cell; PRPF, pre-mRNA processing factor; WISH, whole-mount *in situ* hybridization.

Next, we attempted to explore the potential role of Prpf31 in definitive hematopoiesis, as *prpf31* expression prominently decreased in the CHT at 48 hpf and 3 dpf in *prpf31*^−/−^ zebrafish (Fig. 1, *I*–*L* and *I’*–*L’*). WISH was used to detect the status of definitive HSPCs and its multiple differentiated lineages. At 3 dpf, the expression of HSPC markers (*runx1* and *cmyb*) were dramatically decreased in the CHT of *prpf31*^−/−^ zebrafish, and this insufficiency can be partially rescued by forced expression of a WT *prpf31* mRNA (Fig. 2, *A* and *E*). Meanwhile, the expression of the neutrophils marker *lyz*, the monocytes/macrophages marker *l-plastin*, granulocyte cell marker *mpx*, and the staining signal of Sudan black B labeled neutrophils were also significantly reduced in the CHT of *prpf31*^−/−^ zebrafish at 3 dpf (Fig. 2, *B* and *E*). The expression of the erythrocyte markers *hbae1.1*, *hbae3*, and *hbbe1* were almost undetectable in *prpf31*^−/−^ zebrafish at 4 dpf, despite slightly reduced at 3 dpf (Fig. 2, *C* and *E*). The expression of the early T cell marker *rag1* was almost absent in the thymus of *prpf31*^−/−^ zebrafish (Fig. 2, *D* and *E*). Besides, live imaging of *prpf31*^−/−^ zebrafish showed less flowing cells in blood circulation at 3 dpf (Video S3 and S4). Collectively, these results demonstrate that *prpf31*^−/−^ zebrafish manifest defects in definitive HSPCs and its multiple differentiated lineages.

### HSPC expansion and maintenance are compromised in the CHT of *prpf31*^−/−^ zebrafish

**Figure 3:**
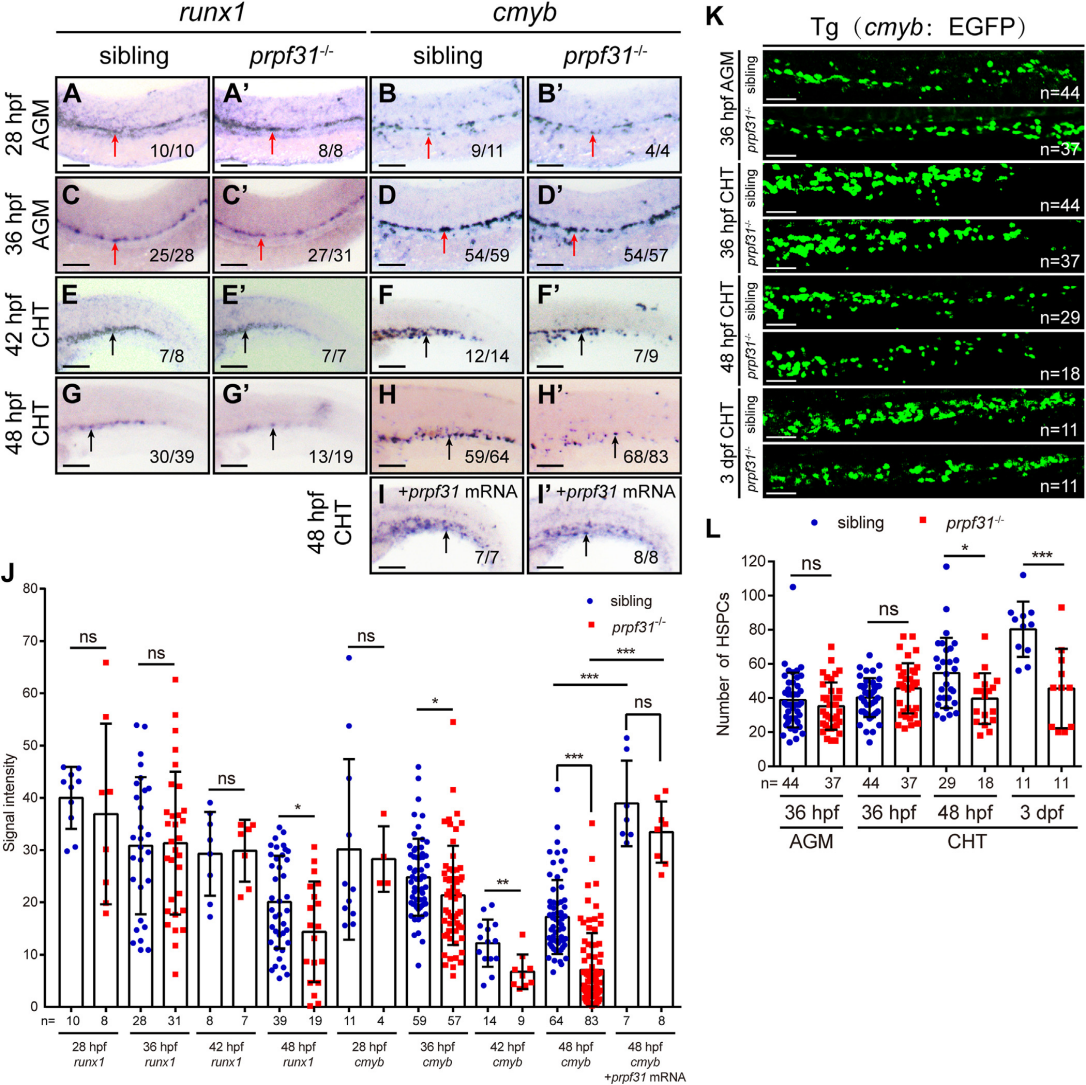
**HSPC expansion and maintenance in the CHT are compromised in *prpf31***^**−/−**^**zebrafish.***A*–*I* and *A*’–*I’*, time-course analysis of the expression of HSPC markers *runx1* and *cmyb* in *prpf31*^−/−^ and sibling embryos from 28 hpf to 48 hpf by WISH. The reduced expression of *cmyb* in *prpf31*^−/−^ zebrafish was rescued by *prpf31* mRNA injection at 48 hpf. Lateral views. *Red arrows* denote the AGM region. *Black arrows* denote the CHT region. The number of embryos with similar gene expression patterns among all embryos examined was shown at the *bottom right* of each panel. The scale bars represent 250 μm. *J*, quantification of mRNA signals of *prpf31*^−/−^ zebrafish and siblings detected by WISH in (*A*–*I* and *A’*–*I’*). The total number of embryos examined was indicated below each column. Mean ± SD; unpaired two-tailed *t* test; ∗*p* < 0.05, ∗∗*p* < 0.01, and ∗∗∗*p* < 0.001; ns, not significant. *K*, *in vivo* observation of HSPCs using transgenic line Tg (*cmyb*: EGFP) of *prpf31*^−/−^ zebrafish and siblings, in the AGM and CHT at 36 hpf, 48 hpf, and 3 dpf. The total number of embryos examined was indicated at the *bottom right* of each panel. The scale bars represent 50 μm. *L*, quantification of the number of HSPCs in the AGM and CHT of *prpf31*^−/−^ zebrafish and siblings observed in (*K*). The total number of embryos examined was indicated below each column. Mean ± SD; unpaired two-tailed *t* test; ∗*p* < 0.05, ∗∗∗*p* < 0.001; ns, not significant. AGM, aorta-gonad-mesonephros; CHT, caudal hematopoietic tissue; dpf, days postfertilization; hpf, hours postfertilization; HSPC, hematopoietic stem and progenitor cell; PRPF, pre-mRNA processing factor; WISH, whole-mount *in situ* hybridization. EGFP, enhanced green fluorescent protein.

In zebrafish definitive hematopoiesis, HSPCs originate directly from the ventral wall of the dorsal aorta through endothelial-to-hematopoietic transition at 26 hpf, and the number of emerging HSPCs peaks at around 36 hpf. Sequentially, these nascent HSPCs enter the circulation through the posterior cardinal vein, and then migrate to and reside in the CHT, a proliferative hematopoietic microenvironment, for pool expansion upon 36 hpf (16, 17, 18, 20). To clarify when defects in HSPCs occur in *prpf31*^−/−^ zebrafish, we performed WISH to analyze the expression of the HSPC markers *runx1* and *cmyb*, at four discrete time points, which cover HSPC emergence in the AGM, migration, and expansion in the CHT. *runx1* expression in the AGM at 28 and 36 hpf, as well as in the CHT at 42 hpf, were almost unperturbed in *prpf31*^−/−^ zebrafish (Fig. 3, *A*, *A*’, *C*, *C’*, *E*, *E’*, and *J*), indicating that the emergence and migration of HSPCs were normal in *prpf31*^−/−^ zebrafish. However, *prpf31*^−/−^ zebrafish displayed reduced *runx1* expression in the CHT at 48 hpf (Fig. 3, *G* and *G’*, and *J*) and 3 dpf (Fig. 2, *A* and *J*). *cmyb* expression in the AGM were also unperturbed at 28 hpf in *prpf31*^−/−^ zebrafish (Fig. 3, B, *B’*, and *J*), while *prpf31*^−/−^ zebrafish showed reduced *cmyb* expression in the AGM at 36 hpf (Fig. 3, *D*, *D’*, and *J*), and such reduction was more profound in the CHT at 42 hpf (Fig. 3, *F*, *F’*, and *J*), 48 hpf (Fig. 3, *H*, *H’*, and *J*), and 3 dpf (Fig. 2, *A* and *J*). This insufficiency can be rescued at 48 hpf by forced expression of a WT *prpf31* mRNA (Fig. 3, *I*, *I’*, and *J*). These results demonstrate that the expansion and maintenance of HSPCs are impaired in the CHT of *prpf31*^−/−^ zebrafish. To visualize the HSPC development *in vivo*, we crossed *prpf31*^+/−^ zebrafish with the transgenic line Tg (*cmyb*: EGFP), in which HSPCs could be labeled by enhanced green fluorescent protein (EGFP) (30). By 36 hpf, the number of EGFP-positive cells in the AGM and CHT of *prpf31*^−/−^ zebrafish were comparable with siblings. However, there was a gradual decrease in the number of HSPCs from 48 hpf to 3 dpf, in the CHT of *prpf31*^−/−^ zebrafish (Fig. 3, *K* and *L*). Taken together, these findings suggest that neither the emergence of the HSPCs in the AGM nor their migration and localization to the CHT are affected, but their transitory expansion and maintenance in the CHT are impaired in *prpf31*^−/−^ zebrafish.

Hematopoiesis and vasculogenesis are tightly associated with each other. Defects in vasculogenesis and patterning result in deficiencies in HSPC emergence, migration, expansion, and maintenance (16, 17, 31, 32). As *prpf31* expression was almost absent in the intersegmental blood vessel and dorsal longitudinal anastomotic vessel of *prpf31*^−/−^ zebrafish at 3 dpf (Fig. 1, *J*, *L*, *J’*, and *L’*), we considered whether defects in vasculogenesis and the vasculature, account for the definitive HSPC deficiencies observed in *prpf31*^−/−^ zebrafish. WISH results showed that the expression of the artery vessel marker *efnb2a* at 24 hpf, and the pan-endothelial cell marker *flk1* at 36 hpf were intact in *prpf31*^−/−^ zebrafish (Fig. S4, *A*, *A’*, *B*, and *B’*). In addition, *in vivo* observation on the vasculature of *prpf31*^−/−^; Tg (*flk1*: EGFP) zebrafish indicated that the overall vasculature and caudal vein plexus in the CHT were well organized at 48 hpf (Fig. S4, *C* and *C’*). Therefore, vasculogenesis and vasculature are not responsible for the observed defects in definitive HSPC expansion and maintenance in *prpf31*^−/−^ zebrafish.

### HSPC proliferation in the CHT of *prpf31*^−/−^ zebrafish is arrested in M phase

**Figure 4:**
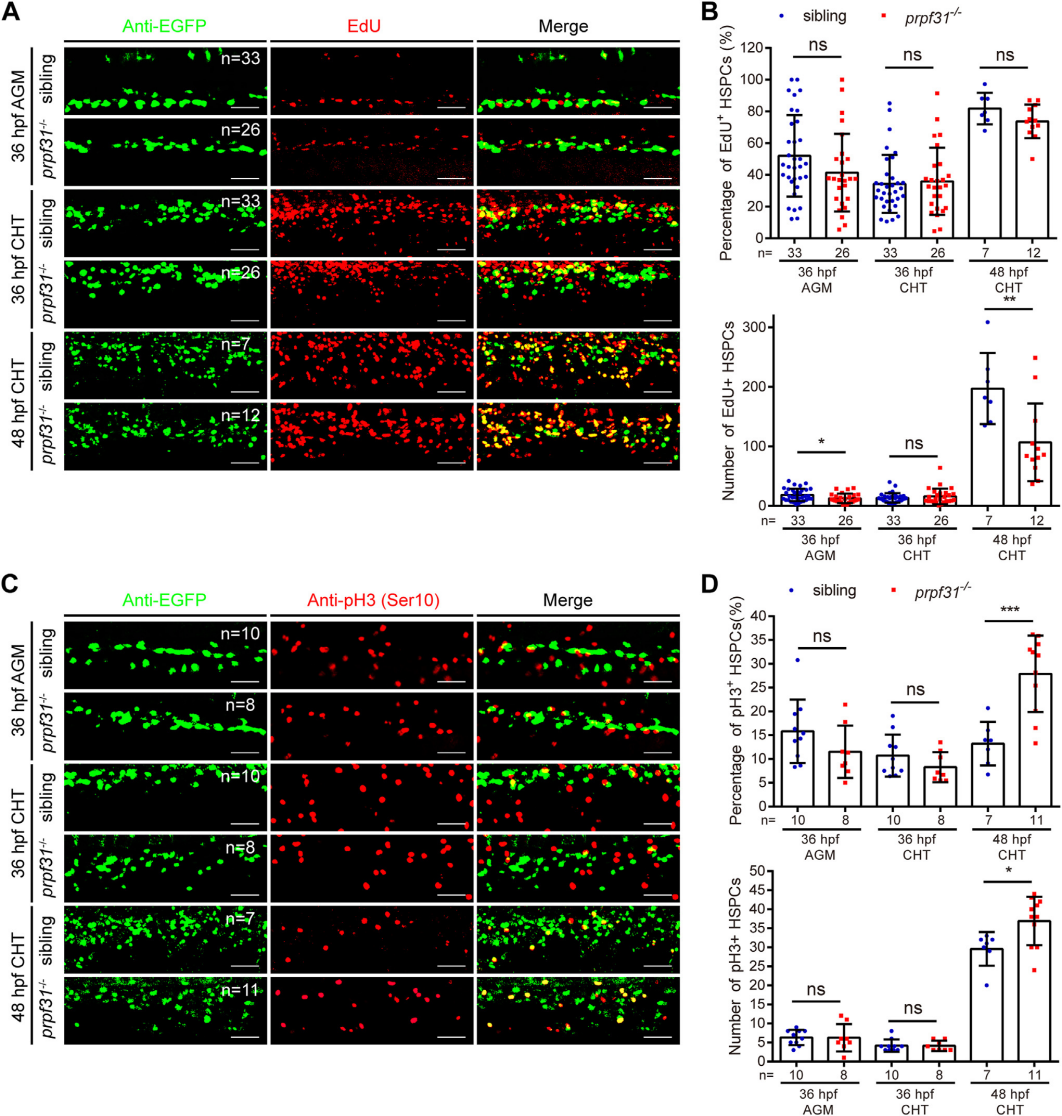
**HSPC proliferation in the CHT of *prpf31***^**−/−**^**zebrafish is arrested in M phase.***A*, double staining of *cmyb*:EGFP and EdU showed no obvious difference of EdU^+^ HSPCs in the AGM and CHT at 36 and 48 hpf. The total number of embryos examined were indicated at the *top right* of each panel. The scale bars represent 50 μm. *B*, quantification of the percentage and number of EdU^+^ HSPCs was detected in (*A*). The total number of embryos examined was indicated below each column. Mean ± SD; unpaired two-tailed *t* test; ∗*p* < 0.05, ∗∗*p* < 0.01; ns, not significant. *C*, double immunostaining of *cmyb*:EGFP and pH3 (Ser10) showed a significant increase of pH3^+^ HSPCs in the CHT at 48 hpf, but not in the AGM and CHT at 36 hpf. The total number of embryos examined were indicated at the *top right* of each panel. The scale bars represent 50 μm. *D*, quantification of the percentage and number of pH3^+^ HSPCs was detected in (*C*). The total number of embryos examined was indicated below each column. Mean ± SD; unpaired two-tailed *t* test; ∗*p* < 0.05, ∗∗∗*p* < 0.001; ns, not significant. AGM, aorta-gonad-mesonephros; CHT, caudal hematopoietic tissue; EdU, 5-ethynyl-29-deoxyuridine; hpf, hours postfertilization; HSPC, hematopoietic stem and progenitor cell; pH3, phospho-histone 3. EGFP, enhanced green fluorescent protein.

To address how Prpf31 deficiency affected the expansion and maintenance of HSPCs in the CHT, we monitored cell death of HSPCs in *prpf31*^−/−^; Tg (*cmyb*: EGFP) zebrafish by TUNEL. No detectable increase in the percentage or number of TUNEL-positive HSPCs were observed in the AGM and CHT from 36 hpf to 48 hpf (Fig. S5, *A* and *B*). To explain the drastic reduction of the HSPCs in the CHT of *prpf31*^−/−^ zebrafish, we further checked the HSPC proliferation status within *prpf31*^−/−^; Tg (*cmyb*: EGFP) zebrafish. The 5-ethynyl-29-deoxyuridine (EdU) incorporation assay showed no significant difference in the percentage of EdU^+^ HSPCs between siblings and *prpf31*^−/−^ zebrafish, in the AGM and CHT from 36 to 48 hpf; although the total number of EdU^+^ HSPCs were significantly reduced in the CHT of *prpf31*^−/−^ zebrafish at 48 hpf (Fig. 4, *A* and *B*). Strikingly, double immunostaining of EGFP and phospho-histone 3 (pH3) Ser10 revealed a noticeable increase in the percentage and number of pH3^+^ HSPCs in the CHT of *prpf31*^−/−^ zebrafish at 48 hpf, whereas no distinguishable difference was detected in the AGM or CHT at 36 hpf (Fig. 4, *C* and *D*). Altogether, these results suggest that HSPCs in the CHT of *prpf31*^−/−^ zebrafish at 48 hpf exhibit cell cycle arrest in M phase.

### Knockdown of *PRPF31* in HEK293 cells inhibited cell proliferation and caused G2/M phase arrest

The effect of PRPF31 on cell cycle progression was also investigated in HEK293 cells. Knockdown of *PRPF31* in HEK293 cells by siRNA showed significant decrease in the expression of PRPF31 protein (Fig. S6*A*). Cell proliferation assay using the Cell Counting Kit-8 (CCK-8) kit indicated significant inhibition of cell proliferation in si-*PRPF31* groups (Fig. S6*B*), which is consistent with the compromised expansion and maintenance of HSPCs observed in *prpf31*^−/−^ zebrafish. Further, flow cytometry analysis of the cell cycle showed more cells exhibited G2/M phase arrest (34.89% of cells) in si-*PRPF31* groups than non-target control (NC) groups (22.02% of cells) (Fig. S6*C*), which is consistent with the cell cycle arrest in M phase observed in *prpf31*^−/−^ HSPCs. The obvious increase in the expression of pH3 (Ser10) protein in si-*PRPF31* groups also consolidated this result (Fig. S6*A*). These results indicated that PRPF31 deficiency inhibits cell proliferation by G2/M arrest.

### *prpf31*^−/−^ zebrafish exhibit severe aberrant AS of mitosis-related genes

**Figure 5:**
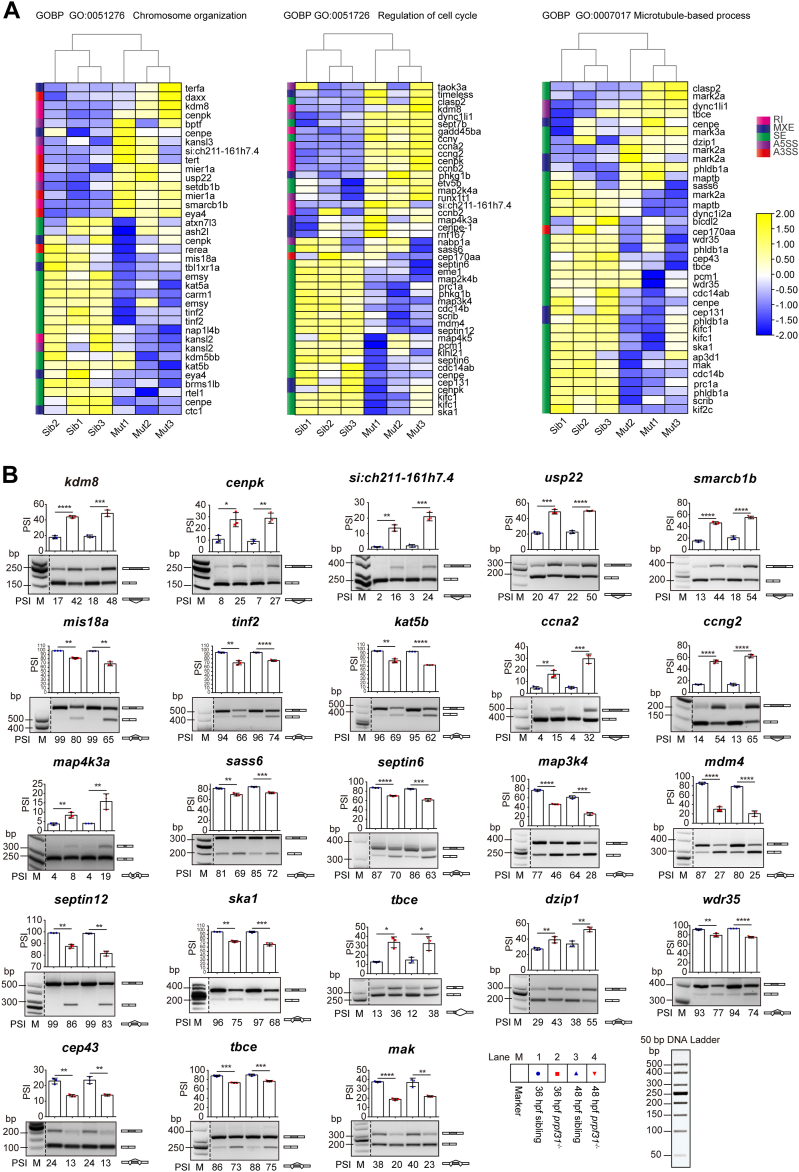
***prpf31***^**−/−**^**zebrafish exhibit severe aberrant alternative splicing of mitosis-related genes.***A*, DASEs corresponding to three representative GOBP terms, that is chromosome organization (GO: 0051276), regulation of cell cycle (GO: 0051726), and microtubule-based process (GO: 0007017) were visualized by heatmaps of normalized PSI. *B*, the DASEs of the representative genes were confirmed by SqRT-PCR. Data were shown as mean ± SD of three independent experiments (n = 3); unpaired two-tailed *t* test; ∗*p* < 0.05, ∗∗*p* < 0.01, ∗∗∗*p* < 0.001, and ∗∗∗∗*p* < 0.0001. DASE, differential alternative splicing event; PRPF, pre-mRNA processing factor; PSI, percent spliced in; SqRT-PCR, semiquantitative reverse transcription PCR. GOBP, Gene Ontology biological processes.

To further elucidate the molecular mechanisms underlying HSPC abatement upon Prpf31 depletion, we profiled the transcriptomes of siblings and *prpf31*^−/−^ zebrafish at 36 hpf by RNA-seq. As Prpf31 functions in RNA splicing, we focused on the differential AS events (DASEs) enriched upon Prpf31 depletion. We identified 793 DASEs in 636 genes across all splicing classes (Dataset S1) of which most (62.92%) were skipped exon events. Most of these skipped exon events (80.16%) showed decreases in percent spliced in (PSI) levels following Prpf31 depletion (Fig. S7*A*). Functional annotation analysis of the differential AS genes revealed that several mitosis-related Gene Ontology (GO) terms were overrepresented (Fig. S7*B*, red boxes; and Datasets S2–S4). DASEs enriched in three representative Gene Ontology biological processes terms, that is, chromosome organization (GO: 0051276), regulation of cell cycle (GO: 0051726), and microtubule (MT)-based process (GO: 0007017), were visualized by heatmaps of normalized PSI (Fig. 5*A* and Dataset S5). The DASEs of the three representative Gene Ontology biological processes terms were confirmed by semiquantitative RT-PCR at 36 and 48 hpf (Fig. 5*B* and Dataset S6). Therefore, we propose that Prpf31 controls HSPC expansion, probably by modulating the AS of mitosis-related genes.

### Aberrant AS of mitosis-related genes leads to mitotic malformations of HSPCs in the CHT of *prpf31*^−/−^ zebrafish

**Figure 6:**
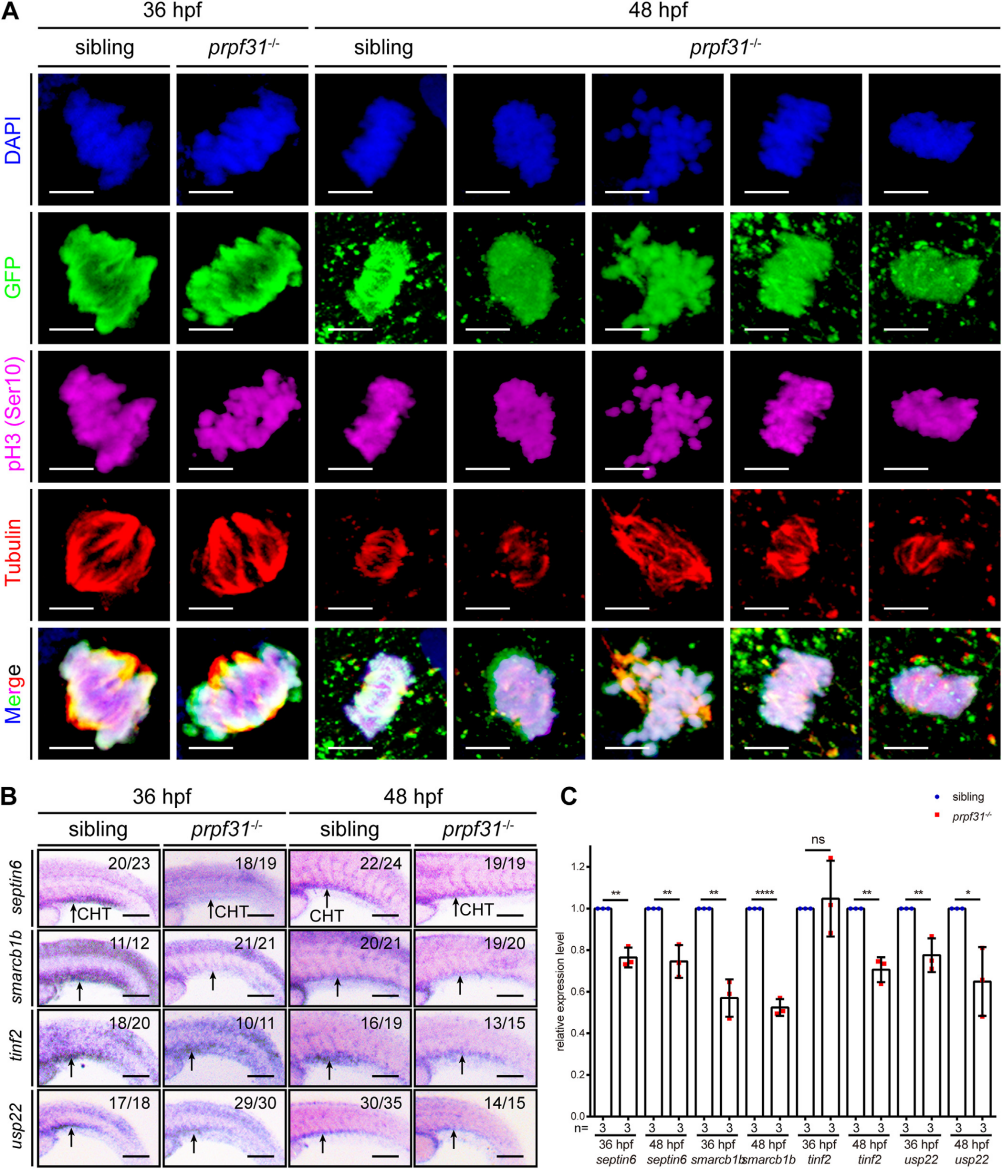
**Aberrant alternative splicing of mitosis-related genes leads to mitotic malformations of HSPCs in the CHT of *prpf31***^**−/−**^**zebrafish.***A*, quadruple staining of DAPI, *cmyb*:EGFP, pH3 (Ser10), and tubulin showed the mitotic status of HSPCs in the CHT of *prpf31*^−/−^ zebrafish and siblings at 36 and 48 hpf. At least 25 mitotic HSPCs in more than six embryos were observed for each group. The scale bars represent 4 μm. *B*, expression of four aberrantly spliced mitosis-related genes (*septin6*, *smarcb1b*, *tinf2*, and *usp22*) in the CHT of siblings and *prpf31*^−/−^ zebrafish at 36 and 48 hpf by WISH. Lateral views. *Black arrows* denote the CHT region. The number of embryos with similar gene expression patterns among all embryos examined were shown on the *top right* of each panel. The scale bars represent 100 μm. *C*, qRT-PCR analysis of four aberrantly spliced mitosis-related genes (*septin6*, *smarcb1b*, *tinf2,* and *usp22*) in siblings and *prpf31*^−/−^ zebrafish at 36 and 48 hpf. Data were shown as mean ± SD of three independent experiments (n = 3); unpaired two-tailed *t* test; ∗*p* < 0.05, ∗∗*p* < 0.01, ∗∗∗∗*p* < 0.0001; ns, not significant. CHT, caudal hematopoietic tissue; hpf, hours postfertilization; HSPC, hematopoietic stem and progenitor cell; pH3, phospho-histone 3; qRT-PCR, quantitative real-time PCR; WISH, whole-mount in situ hybridization. DAPI, 4′,6-diamidino-2-phenylindole; EGFP, enhanced green fluorescent protein.

Next, we examined the mitotic status of HSPCs in the CHT of *prpf31*^−/−^; Tg (*cmyb*: EGFP) zebrafish. The mitotic HSPCs in the CHT of *prpf31*^−/−^ zebrafish exhibited typical mitotic prometaphase morphology at 36 hpf (Fig. 6*A*). The chromosomes were condensed and aligned, and the spindle MTs were well-organized. However, abnormal mitotic prometaphase morphology was observed at 48 hpf. The chromosomes exhibited oscillations and dispersed along the spindle MTs. The spindle MTs were disorganized, with shortened, elongated, monopolar, or polypolar spindle MTs (Fig. 6*A*). These defects are in agreement with morphological mitotic abnormalities observed in PRPF31-deficient human cells (33). Moreover, four genes (*septin6*, *smarcb1b*, *tinf2*, and *usp22*), which were mitosis-related and aberrantly spliced (Fig. 5, *A* and *B*), have been associated with the pathologies of specific HMs (34, 35, 36, 37, 38). These four genes were prominently expressed in the CHT at 36 and 48 hpf as shown by WISH, with their expression decreased in the CHT of *prpf31*^−/−^ zebrafish at 48 hpf (Fig. 6*B*). We also detected reduced expression of these four genes by quantitative real-time PCR (qRT-PCR) (Fig. 6*C*). Collectively, we demonstrate that aberrations in AS of mitosis-related genes, contribute to the mitotic malformations, M phase arrest, and expansion impairment, in the HSPCs of *prpf31*^−/−^ zebrafish.

## Discussion

**Figure 7:**
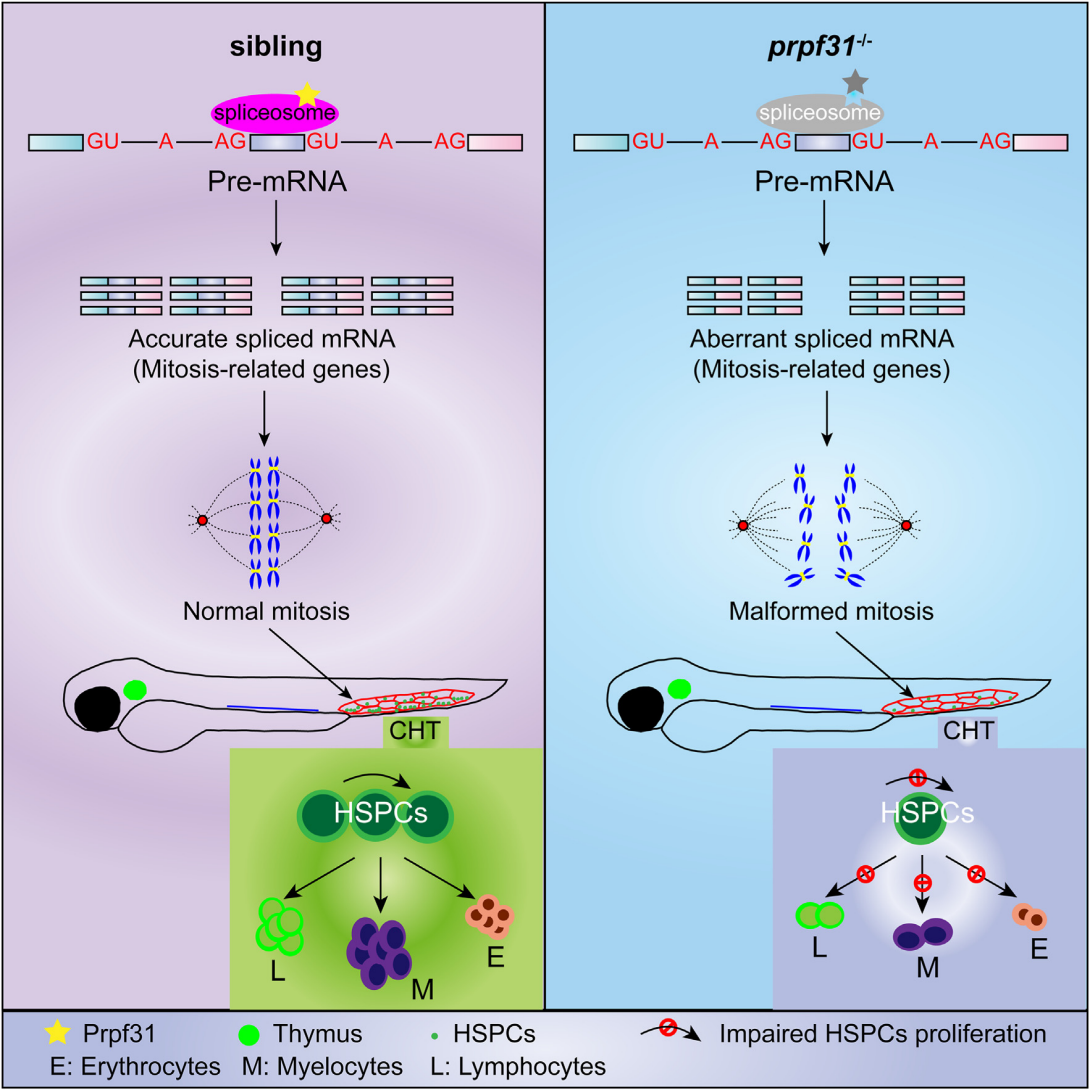
**Model of Prpf31 action in HSPC expansion during zebrafish embryogenesis.** In zebrafish embryogenesis, the nascent HSPCs undergo extensive proliferation for pool expansion and commitment-proliferation-differentiation in the CHT, which demand rapid and periodic regulated pre-mRNA alterative splicing. Accurate and sequential regulated pre-mRNA alterative splicing ensure efficient and precise HSPCs mitosis for rapid blood replenishment. In *prpf31*^−/−^ zebrafish, the spliceosomes cannot assemble effectively due to deficiency of Prpf31, which compromises the splicing efficiency of the spliceosome machine, and results in aberrant mRNA alternative splicing, perturbs the alternative splicing of mitosis-related genes, predisposes HSPCs to malformed mitosis and cell cycle arrest in M phase, and eventually impaired the expansion and differentiation of HSPCs in the CHT. CHT, caudal hematopoietic tissue; HSPC, hematopoietic stem and progenitor cell; PRPF, pre-mRNA processing factor.

PRPF31 is a ubiquitously expressed splicing factor, and plays a crucial role in the assembly of the spliceosome. Heterozygous mutations in *PRPF31* cause adRP (39, 40). It is fascinating why mutations in this ubiquitously expressed splicing factor, specifically result in clinical phenotype restricted to the retina. Here, for the first time, we report that Prpf31 plays a pivotal role in HSPC expansion during zebrafish embryogenesis. Deprivation of Prpf31 in zebrafish inhibited the expansion of HSPCs by perturbing the AS of mitosis-related genes (Fig. 7).

AS is particular universal in the nervous and immune systems in which cells undergo rapid cell proliferation, differentiation, and migration (41). Our preliminary study demonstrated that Prpf31 deficiency compromised the mitosis and differentiation of retinal progenitor cells, by interfering with the AS of genes involved in spindle organization and DNA repair (29). In current study, we elucidate, for the first time, that depletion of Prpf31 impairs HSPC expansion by perturbing the AS of mitosis-related genes. The two above studies exactly delineate the sensitivity of neuron and immune system to aberrant AS, respectively. In embryonic definitive hematopoiesis, HSPCs undergo substantial expansion for self-renewal and commitment-proliferation-differentiation in the fetal liver (CHT in zebrafish) (42). These extensively proliferating HSPCs demand rapid and periodic regulated RNA transcription and protein synthesis, to achieve fast and effective blood replenishment, which are orchestrated by a series of biological processes, including pre-mRNA AS (43, 44). More than a thousand genes undergo accurate and efficient periodic AS during cell cycle progression, which highlights a critical role for AS in mitosis (45, 46). Prominently, fetal liver (CHT in zebrafish) HSPCs, with a high proliferative rate, display enhanced sensitivity to splicing factor mutations (21, 22, 24). This is in accordance with the perturbations in AS of mitosis-related genes and the deficiency in HSPC proliferation observed in *prpf31*^−/−^ zebrafish.

By using high-throughput transcriptomic analyses, researchers have found extensive and dynamic AS program takes place during human hematopoietic stem cell differentiation. AS manifests complex lineage and differentiation stage specificity during hematopoietic development. Moreover, spliceosomal components also show dynamic and tightly controlled expression during different hematopoietic differentiation stages (47, 48). We searched the expression pattern of *prpf31* in iCHTatlas, a web server providing spatiotemporal and high-resolution transcriptomic information of genes that are expressed in the CHT region during zebrafish HSPC expansion (28). We found that *prpf31* manifests relatively higher expression in HSPCs at 52 hpf (approximately the initial period of HSPC expansion), than the other hematopoietic stages (28 hpf, 36 hpf，3 dpf, 4 dpf, and 3 mpf), which suggests higher demand of Prpf31 during the initial period of HSPC expansion. Therefore, HSPCs may be more sensitive to the decrease of Prpf31 during HSPC expansion.

Approximately 95% of human multiexon genes undergo AS, but not all alternatively spliced transcripts produce functional proteins (2). Majority of all alternatively spliced transcripts harbor premature termination codons. These transcripts are predicted to be selectively degraded by the nonsense-mediated mRNA decay (NMD) pathway, thereby preventing the accumulation of such transcripts and production of potentially aberrant proteins (49). Numerous studies have reported a coupling action of AS to NMD (AS-NMD). This coordinated action is considered to control the ratio of functional to nonfunctional mRNA transcripts, ultimately degrading non-functional mRNAs containing premature termination codons (50). Autoregulation of splicing factors by AS-NMD have been documented in classical SR proteins (such as SRSF1, SRSF3, SRSF4, SRSF5, or SRSF7), hnRNP proteins (such as PTBP1, hnRNPA2B1, or hnRNPL), and spliceosome components (such as small nuclear ribonucleoprotein polypeptides B and B1), etc. (50). Ying Ge *et al.* (51) have summarized the functional consequences of AS within the hematopoietic system by coupling it to NMD. Accordingly, it is possible that Prpf31 may affect the AS of critical mitosis-related genes by the coupling action of AS to NMD (AS-NMD) in the same way as other splicing factors.

Currently, with the development of massively parallel sequencing technologies, a number of gene mutations in spliceosomal components have been implicated in the pathogenesis of human HMs. However, the intrinsic pathogenesis of spliceosomal components participating in HMs remains to be investigated (5, 12). The COSMIC database shows 0.22% (16/7278) of *PRPF31* mutation frequency in patients with HMs. Our study, therefore, may provide mechanistic insights for the clinical investigations of *PRPF31* mutations in HMs. HSPCs in *prpf31*^−/−^ zebrafish exhibited M phase arrest and abnormal mitotic prometaphase morphology. Experiments carried out by other researchers demonstrated that splicing factor *SRSF2* mutations induced dramatic inhibition of proliferation and G2/M phase arrest in primary human CD34+ cells and HEK293T cells (52, 53), while these deleterious effects did not occur in *SRSF2* mutated K562 cells or SRSF2-P95H myelodysplastic syndromes mouse models (52, 54, 55). Therefore, this G2/M arrest appears to occur specifically in the clonal hematopoiesis of indeterminate potential or preleukemic conditions. Disclosing the triggers that allow G2/M arrested cells reenter into the cell cycle may help to clarify the causes of malignant transformation of HMs and may shed light on potential combinational therapeutic targets.

We demonstrated that four mitosis-related genes (*septin6*, *smarcb1b*, *tinf2*, and *usp22*) were aberrantly spliced, with their expression decreased in the CHT of *prpf31*^−/−^ zebrafish. Additionally, we found that these four genes were associated with pathologies of specific HMs. SEPTIN6 has been identified as a myeloid/lymphoid leukemia fusion partner (34). Loss of SMARCB1 has been shown to promote acute myeloid leukemia cell migration and survival (35). Deletions of SMARCB1 are frequent in patients with chronic myeloid leukemia (36). *TINF2* is mutated in patients with dyskeratosis congenita, which is a heterogeneous inherited bone marrow failure syndrome (38). USP22 deficiency in Ras-driven myeloproliferative neoplasm blocks myeloid differentiation promoting acute myeloid leukemia (37). These studies further support the involvement of PRPF31 in normal hematopoiesis and HMs.

In summary, our research reveals a previously unrecognized role of Prpf31 in regulating HSPC expansion. Disclosing the AS regulatory function of Prpf31 in hematopoietic development might be helpful to clarify the physiological and pathological mechanisms of splicing factor participating in the hematopoietic development process.

## Experimental procedures

### Ethics statement

All zebrafish maintenance procedures and experiments were conducted under the guidance and approval of the Animal Research Ethics Committee of Tongji Medical College, Huazhong University of Science and Technology (Approval ID 2019-S907).

### Zebrafish maintenance and manipulation

The CRISPR/Cas9-mediated generation of *prpf31*^−/−^ zebrafish was described in our previous paper (29). WT siblings represent *prpf31*^+/+^ zebrafish bred from male and female *prpf31*^+/−^ parents. The *prpf31*^+/−^; Tg (*cmyb*: EGFP) and *prpf31*^+/−^; Tg (*flk1*: EGFP) transgenic zebrafish strains were obtained from crossing of *prpf31*^+/−^ zebrafish with the WT; Tg (*cmyb*: EGFP) and WT; Tg (*flk1*: EGFP) transgenic lines, respectively.

### RNA extraction, complementary DNA synthesis, qRT-PCR, and SqRT-PCR

Total RNA was isolated from dissected tissues (for zebrafish at 24 hpf, containing the RBI and ICM regions; for zebrafish at 36 hpf, 48 hpf, and 3 dpf, containing the AGM and CHT regions) (n ≥ 30 for each group) of zebrafish using Total RNA Extraction Reagent (Vazyme, R401-01). Complementary DNA was synthesized from 1 μg of total RNA using HiScript II Q RT SuperMix for qPCR (Vazyme, R223-01). The qRT-PCR was performed with AceQ qPCR SYBR Green Master Mix (Vazyme, Q141-03) in a StepOnePlus real-time PCR system (Life Technologies). Semiquantitative reverse transcription PCR (SqRT-PCR) was performed using Green Taq Mix (Vazyme, P131-01). The abundance of the SqRT-PCR segment bands was measured using Image J (https://imagej.net/ij/). Primers used for qRT-PCR and SqRT-PCR are summarized in Dataset S7.

### Protein extraction and immunoblotting analysis

For zebrafish at 24 hpf, the tails of zebrafish embryos were dissected for genotyping, the residual tissues (containing the primitive hematopoietic RBI and ICM regions) (n ≥ 30 for each group) with similar genotypes were lysed and ultrasonicated in radio immunoprecipitation assay (RIPA) lysis buffer (Beyotime, P0013B). For zebrafish at 36 hpf, 48 hpf, and 3 dpf, the heads of zebrafish embryos were dissected for genotyping, the residual tails (containing the AGM and CHT regions) (n ≥ 30 for each group) with similar genotypes, were lysed and ultrasonicated in RIPA lysis buffer.

For HEK293 cells, cells were digested with trypsin, washed with PBS, lysed, and ultrasonicated in RIPA lysis buffer after 48 h of transfection.

Western blotting was performed as described previously (56). The following primary antibodies were used: anti-PRPF31 antibody (GeneTex, GTX117081, 1:1000), anti-GAPDH antibody (Proteintech, 60004-1-Ig, 1:3000), and anti-phospho-histone H3 (Ser10) antibody (Affinity, AF3358, 1:1000).

### Whole-mount *in situ* hybridization

Primers used for probe synthesis are listed in Dataset S7. The amplificated segments were ligated into the pGEM-T easy vector (Promega, A137A). The linearized plasmids were transcribed *in vitro* by T7 or SP6 RNA polymerase (Promega, P207E, P108B) with digoxigenin RNA Labeling Mixture (Roche, 11277073910). WISH was carried out using conventional 4-nitro blue tetrazolium chloride/5-bromo-4-chloro-3-indolyl-phosphate precipitation by alkaline phosphatase (Roche, 11681451001) (57). Images were captured by a series zoom stereo microscope (Cnoptec, SZ680) connected with a digital camera. After imaging, DNA of the embryos was extracted for genotyping. Signal intensity of mRNA level detected by WISH was quantified as previous described (58).

### Sudan black B staining and O-dianisidine staining

Sudan Black B and O-dianisidine staining were performed as described previously (59, 60).

For Sudan black staining, embryos at indicated stages were fixed with 4% paraformaldehyde (PFA) at 4 °C overnight. After rinsing in phosphate buffered saline with tween 20 (PBST), the embryos were incubated with Sudan black B (Sigma-Aldrich, 199664) working solution for 30 min at room temperature, washed extensively in 70% ethanol, and then progressively rehydrated to PBST. Finally, the embryos were transferred to 100% glycerol, and imaged by a series zoom stereo microscope (Cnoptec, SZ680) connected with a digital camera.

For O-dianisidine staining, live embryos at indicated stages were incubated with O-dianisidine (Sigma-Aldrich, D1943) staining solution for 15 min in the dark, washed with PBST, fixed in 4% PFA for 30 min. After washed with PBST, stained embryos were mounted in 100% glycerol for imaging.

### Live imaging of zebrafish embryos

Zebrafish embryos were collected at indicated stages, and live imaging of blood circulation was recorded using System microscope BX53 (Olympus).

### *In vitro* transcription and microinjection

The phenotypic rescue was conducted as previously described (29). Briefly, we constructed a PCS2+8CmCherry-zf*prpf31* CDS full-length plasmid. Then, the plasmid was linearized and *in vitro* transcribed into mRNA. The mRNA was injected into the 1 to 2 cell stage embryos bred from male and female *prpf31*^+/−^ parents.

### TUNEL labeling, EdU incorporation, and immunostaining

For TUNEL labeling, embryos of Tg (*cmyb*: EGFP) zebrafish were collected at 36 and 48 hpf and fixed in 4% PFA at 4 °C overnight. Next, the embryos were washed, and digested with proteinase K (Tiangen, RT403). Then, the embryos were blocked with blocking buffer, incubated with goat anti-GFP antibody (GeneTex, GTX26673, 1:200), followed by incubating with the secondary antibody. After that, the embryos were labeled with TUNEL BrightRed Apoptosis Detection Kit (Vazyme, A113-01).

For EdU incorporation, embryos of Tg (*cmyb*: EGFP) zebrafish were collected, and immersed in egg water containing 2 mM EdU/10% dimethyl sulfoxide for 30 min at 4 °C. After rinsing several times in egg water, the embryos were transferred into fresh egg water incubating at 28.5 °C for the next 2 h. Then, the embryos were collected, fixed, washed, digested, blocked, and incubated with primary and secondary antibodies as described in the TUNEL labeling assay. After that, the embryos were stained with the Cell-Light EdU Apollo567 *In Vitro* Kit (RiboBio, C10310-1).

For immunostaining, the embryos were collected, fixed, washed, digested, blocked, and incubated with primary and secondary antibodies as aforementioned. Then the embryos were stained with 4′,6-diamidino-2-phenylindole (Beyotime, C1002, 1:1000). The following primary antibodies were used: goat anti-GFP antibody (GeneTex, GTX26673, 1:200), rabbit anti-phospho-histone H3 (Ser10) antibody (Affinity, AF3358, 1:200), and rat anti-tubulin antibody (Abcam, ab6160, 1:300).

The heads of embryos were used for genotyping, and the tails (containing both AGM and CHT) were mounted on slices, and coverslipped with Mowiol mounting media. All images were captured by FV1000 (Olympus).

### RNA-seq and bioinformatics analysis

Embryos, which were bred from male and female *prpf31*^+/−^ parents, were collected at 36 hpf. The heads were dissected for genotyping, the residual dissected tails (including the AGM and CHT) (n ≥ 30 for each group) of similar genotypes were used for RNA extraction. RNA sequencing was performed on an Illumina HiSeq2000 platform by Novogene. The Hisat2 was used to map the RNA-seq data to the zebrafish GRCz11 genome. DASEs were selected using the following cut-off values: false discovery rate <0.05, and |ΔPSI| >0.1. Functional GO enrichment analysis was performed by Metascape (http://metascape.org/gp/index.html), and visualized by bioinformatics (http://www.bioinformatics.com.cn/). Heatmaps were plotted by TBTools.

### Cell culture and transfection

HEK293 cells were cultured according to standard protocols. Cells were seeded into 12-well plates (1 × 10^5^ cells per well). After 20 h, cells were transfected with NC or *prpf31* si-RNA using the Lipofectamine 3000 (Invitrogen, L3000075) according to the manufacturer's protocol. The efficiency of interference was confirmed by Western blotting after 48 h of transfection.

### Cell proliferation assay

The cell proliferation assay was performed using the CCK-8 kit (BioSharp, BS350B) according to the manufacturer's protocol. Briefly, 100 μl cell suspension containing 6 × 10^3^ cells of HEK293 cell lines were seeded into 96-well plates and transfected with NC or *prpf31* si-RNA after 20 h. Subsequently, transfected cells were cultured for the next 24, 48, 72, or 96 h. Then, the culture medium was replaced with 100 μl fresh culture medium and 10 μl CCK-8. After incubation for an additional 2 h, the absorbance was measured at 450 nm using a Microplate Spectrophotometer (BioTek Eon).

### Flow cytometry analysis of the cell cycle

Cell cycle distribution was assessed by flow cytometry. Briefly, 1000 μl cell suspension containing 1 × 10^5^ cells of HEK293 cell lines were seeded into 12-well plates and transfected with NC or *prpf31* si-RNA after 20 h. Subsequently, transfected cells were cultured for the next 48 h. Then, cells were harvested, washed with cold PBS, and fixed using 75% cold ethanol overnight at 4 °C. After centrifugation, the cells were washed twice with cold PBS, and incubated with RNase at 37 °C for 30 min. Subsequently, the cells were stained with propidium iodide staining solution in the dark for 30 min. Cell cycle distribution was analyzed using CytoFLEX Flow Cytometer and CytExpert software (Beckman Coulter; https://www.beckman.com/flow-cytometry/research-flow-cytometers/cytoflex/software).

### Statistical analysis

All experiments were independently performed at least three times (n ≥ 3). Data were analyzed with GraphPad Prism (https://www.graphpad.com/) using unpaired two-tailed Student *t* test and presented as mean ± SD *p* <0.05 was statistically significant (∗*p* < 0.05, ∗∗*p* < 0.01, ∗∗∗*p* < 0.001, and ∗∗∗∗*p* < 0.0001).

## Data availability

RNA-seq data performed for this study is deposited in Gene Expression Omnibus database under the accession number GSE171661.

## Supporting information

This article contains supporting information.

## Conflict of interest

The authors declare that they have no conflicts of interest with the contents of this article.
