# Supplementary material for: The splicing factor Prpf31 is required for hematopoietic stem and progenitor cell expansion during zebrafish embryogenesis
Source: J Biol Chem. 2024 Feb 19;300(3):105772. doi: 10.1016/j.jbc.2024.105772 (PMC10959673; doi:10.1016/j.jbc.2024.105772)
Supplement: Supporting information [file mmc12.docx]

**The splicing factor Prpf31 is required for** **hematopoietic stem and progenitor cell expansion during zebrafish embryogenesis**

**Yuexia Lv^1^****^, 2, ‡^, Jingzhen Li^1, 3, ‡^, Shanshan Yu^1, 4^,** **Yangjun Zhang^5^, Hualei Hu^1^, Kui Sun^1^, Danna Jia^1^, Yunqiao Han^1^, Jiayi Tu^1^, Yuwen Huang****^1^, Xiliang Liu^1^,** **Xianghan Zhang^1^, Pan Gao^1^, Xiang Chen^1^, Mark Thomas Shaw Williams^6^, Zhaohui Tang^1^, Xinhua Shu^6^, Mugen Liu^1, *^, Xiang Ren^1, *^**

*From the ^1^ Key Laboratory of Molecular Biophysics of the Ministry of Education,* *College of Life Science and Technology, Huazhong University of Science and Technology, Wuhan, China**;* *^2^ Department of Prenatal Diagnosis Center, The Third Affiliated Hospital of Zhengzhou University, Zhengzhou, China; ^3^ Research Center for Biochemistry and Molecular Biology, Jiangsu Key Laboratory of Brain Disease Bioinformation, Xuzhou Medical University, Xuzhou, China; ^4^ Institute of Visual Neuroscience and Stem Cell Engineering, College of Life Sciences and Health, Wuhan University of Science and Technology, Wuhan, China; ^5^ Department of Urology, Tongji Hospital, Tongji Medical College, Huazhong University of Science and Technology, Wuhan, China; ^6^ Department of Biological and Biomedical Sciences,* *Glasgow Caledonian University,* *Glasgow, United Kingdom.*

‡These authors contributed equally to this work.

*For correspondence: Xiang Ren, renxiang@hust.edu.cn; Mugen Liu, lium@hust.edu.cn.

**Supporting information**

**Supporting figure**

**
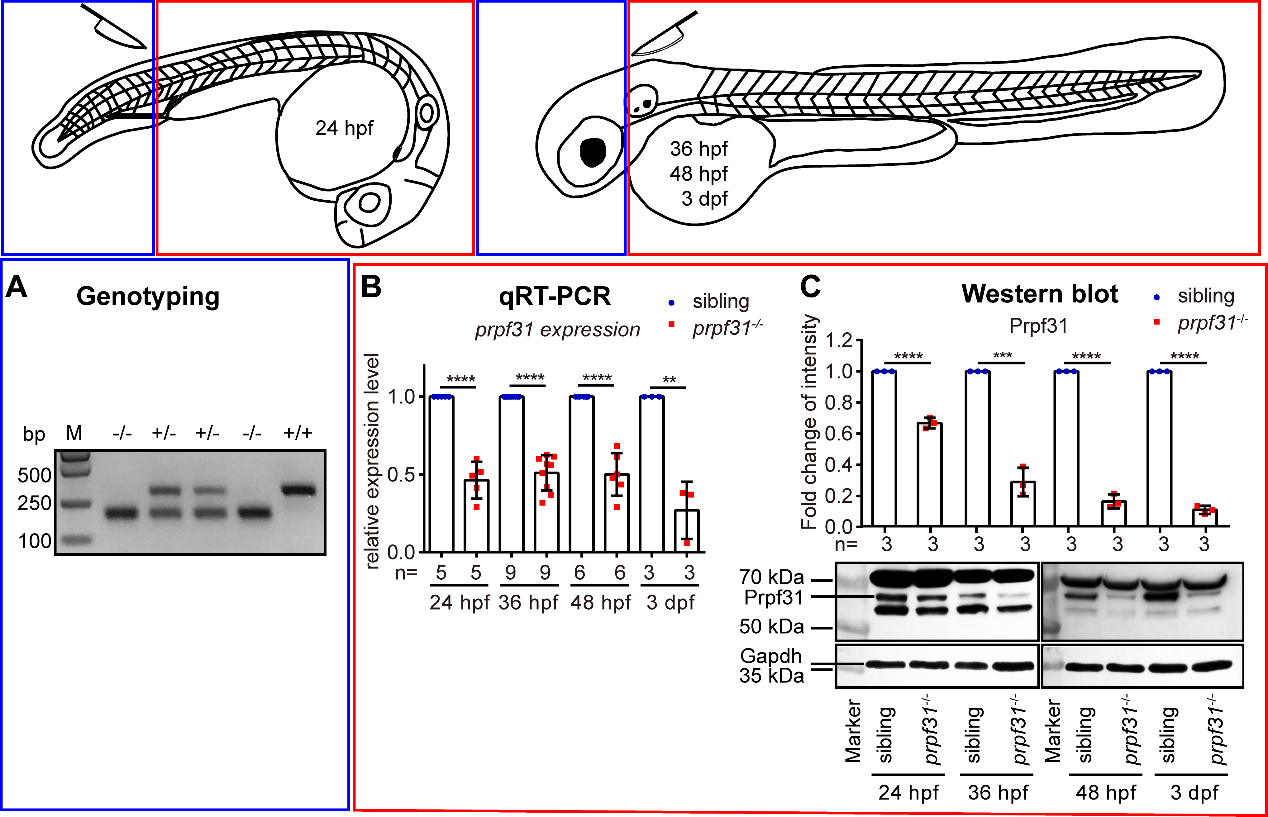
**

**Figure S1. Schematic diagram of tissue dissection of zebraﬁsh used in this study, and genotype, mRNA, and protein level validation.**

(**A**) Amplification and DNA gel electrophoresis of *prpf31* gene segments for genotype validation. (**B**) Reduced *prpf31* mRNA level was detected by qRT-PCR analysis in *prpf31*^-/-^ zebrafish at 24 hpf, 36 hpf, 48 hpf, and 3 dpf. Statistical data of at least three independent experiments were analyzed using unpaired two-tailed *t*-test, and shown as mean ± SD; **p < 0.01, ****p < 0.0001. (**C**) Western blot analysis demonstrated reduced expression of Prpf31 protein in *prpf31*^-/-^ zebrafish at 24 hpf, 36 hpf, 48 hpf, and 3 dpf. Gapdh was used as an internal control. Data were shown as mean ± SD of three independent experiments (n = 3); unpaired two-tailed *t* test; ***p < 0.001, ****p < 0.0001.

**
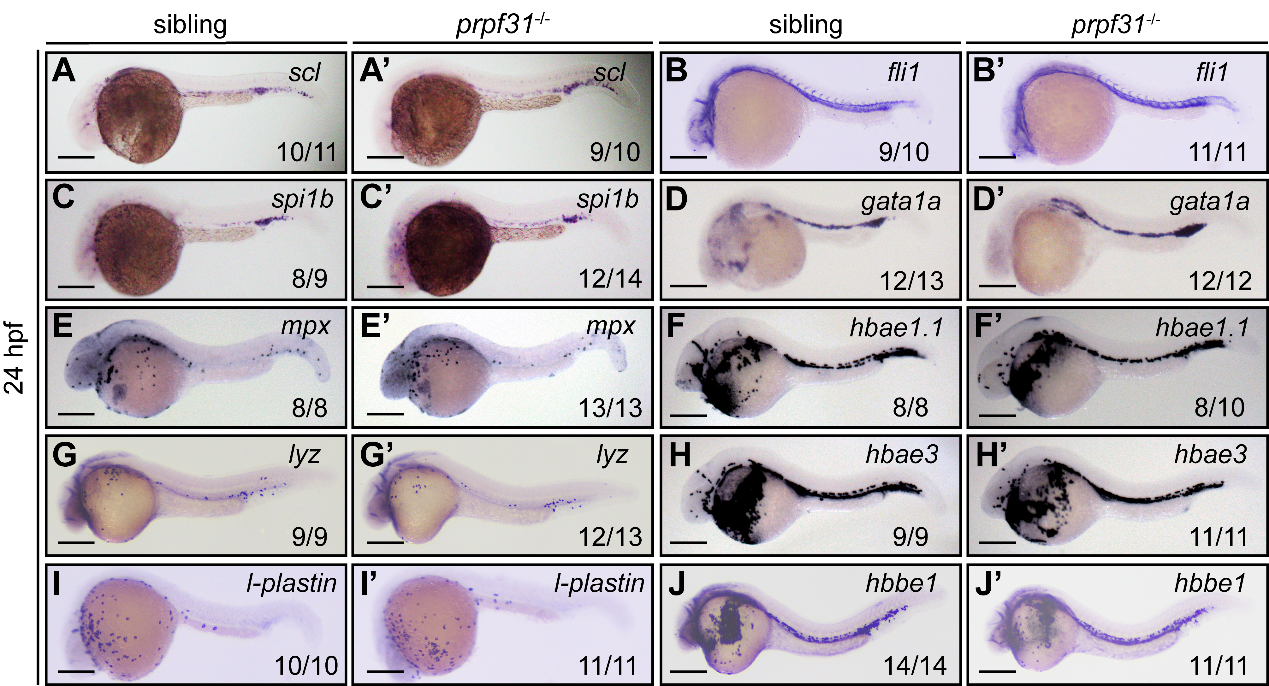
**

**Figure S2. Primitive hematopoiesis is normal in *prpf31*^-/-^ zebrafish.**

(**A-J** and **A’-J’**) Expression of *scl* (A, A’), *fli1* (B, B’), *spi1b* (C, C’), *gata1* (D, D’), *mpx* (E, E’), *hbae1.1* (F, F’), *lyz* (G, G’), *hbae3* (H, H’), *l-plastin* (I, I’), and *hbbe1* (J, J’) were indistinguishable between *prpf31*^-/-^ zebrafish and wild-type siblings at 24 hpf by WISH. Lateral views, anterior to the left, and dorsal upwards. The number of embryos with similar gene expression patterns among all embryos examined were shown at the bottom right of each panel. Scale bars, 250 μm.


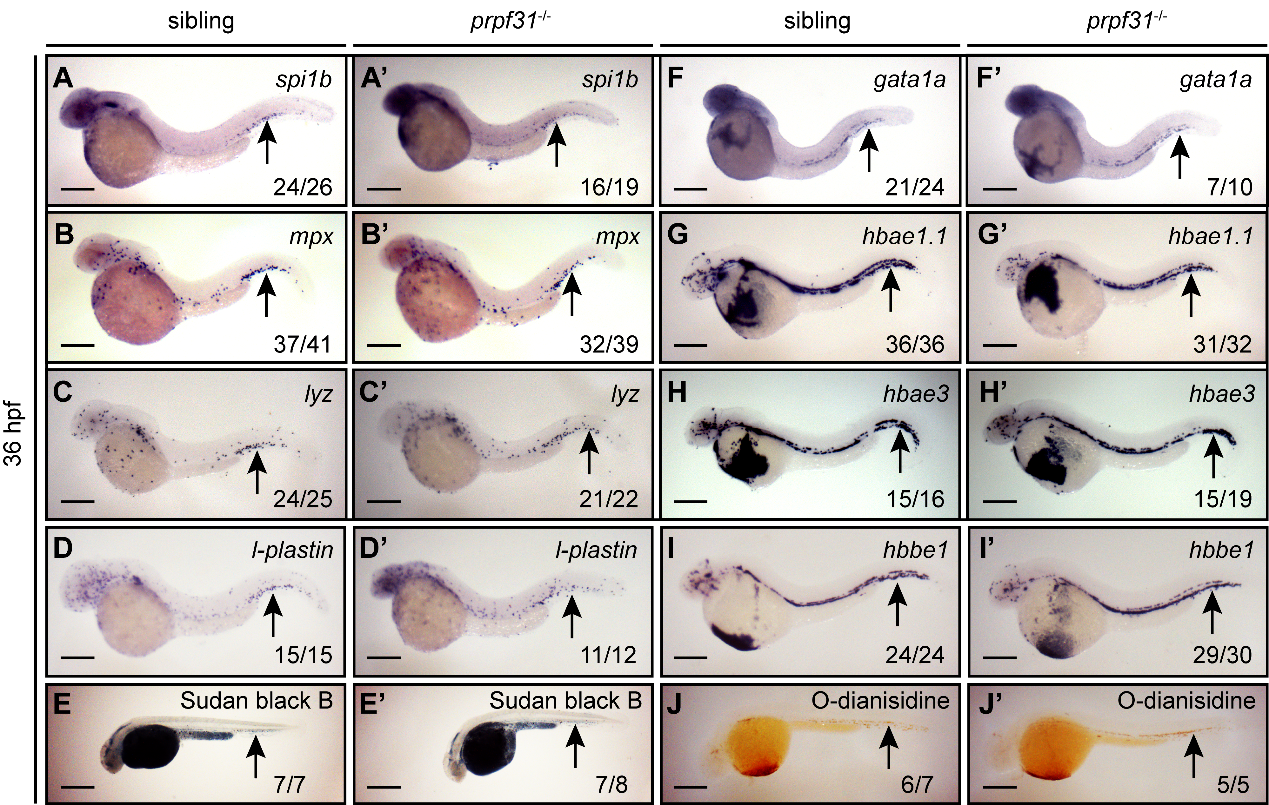


**Figure S3. *prpf31*^-/-^ zebrafish exhibit normal EMP formation.**

(**A-J** and **A’-J’**) Expression of *spi1b* (A, A’), *mpx* (B, B’), *lyz* (C, C’), *l-plastin* (D, D’), *gata1a* (F, F’), *hbae1.1* (G, G’), *hbae3* (H, H’), and *hbbe1* (I, I’), and staining signal of Sudan black B labeled neutrophils (E, E’) and O-Dianisidine labeled erythrocytes (J, J’) were comparable between *prpf31*^-/-^ zebrafish and siblings, at 36 hpf by WISH. Lateral views, anterior to the left, and dorsal upwards. The number of embryos with similar gene expression patterns among all embryos examined were shown at the bottom right of each panel. Scale bars, 250 μm.


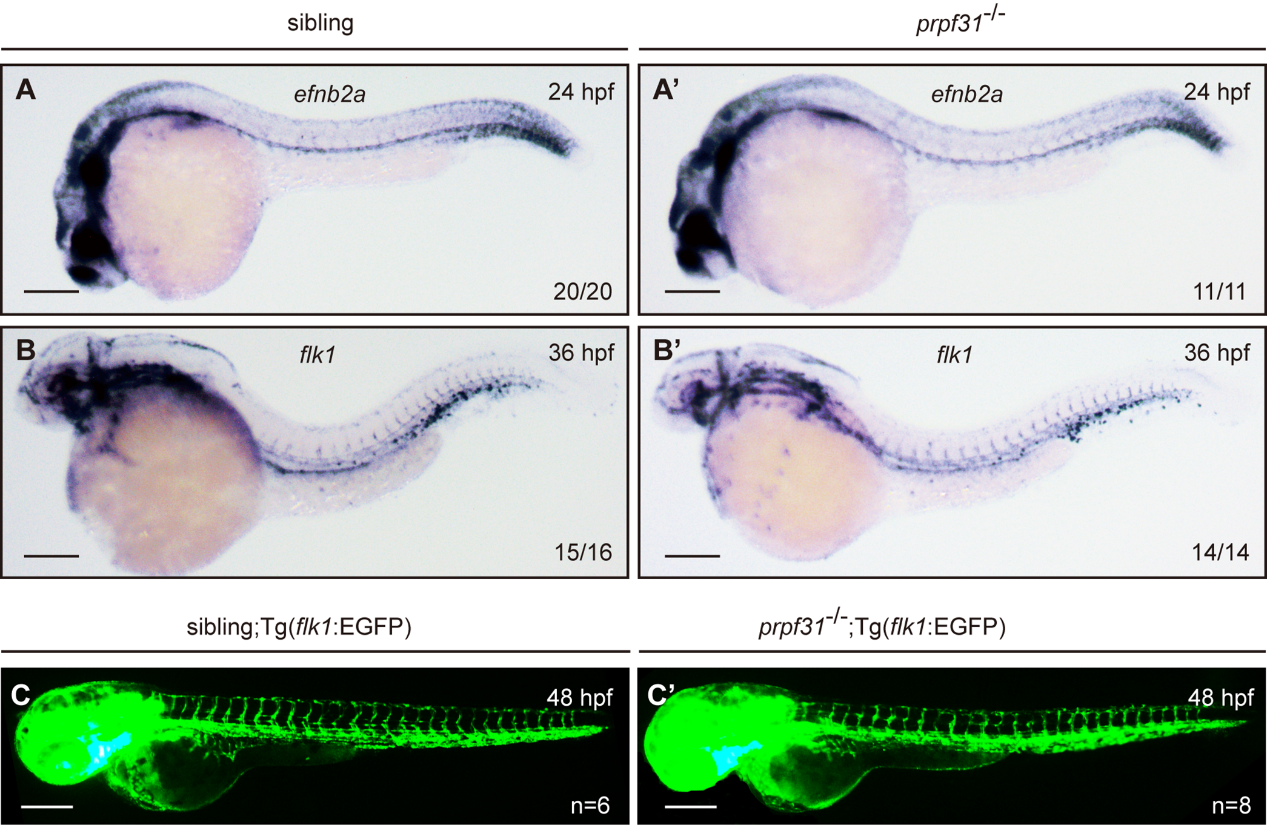


**Figure S4. Vasculogenesis and vasculature are intact in *prpf31*^-/-^ zebrafish.**

(**A-B** and **A’-B’**) WISH results showed normal expression of *efnb2a* (A, A’) and *flk1* (B, B’) in *prpf31*^-/-^ zebrafish and siblings, respectively at 24 and 36 hpf. The number of embryos with similar gene expression patterns among all embryos examined were shown at the bottom right of each panel. Scale bars, 250 μm. (**C** and **C’**) *In vivo* observation of the vasculature within a Tg (*flk1*: EGFP) transgenic background demonstrated well organized vasculature and caudal vein plexus in the CHT in both *prpf31*^-/-^ zebrafish and siblings at 48 hpf. The total number of embryos examined were indicated at the bottom right of each panel. Scale bars, 250 μm.


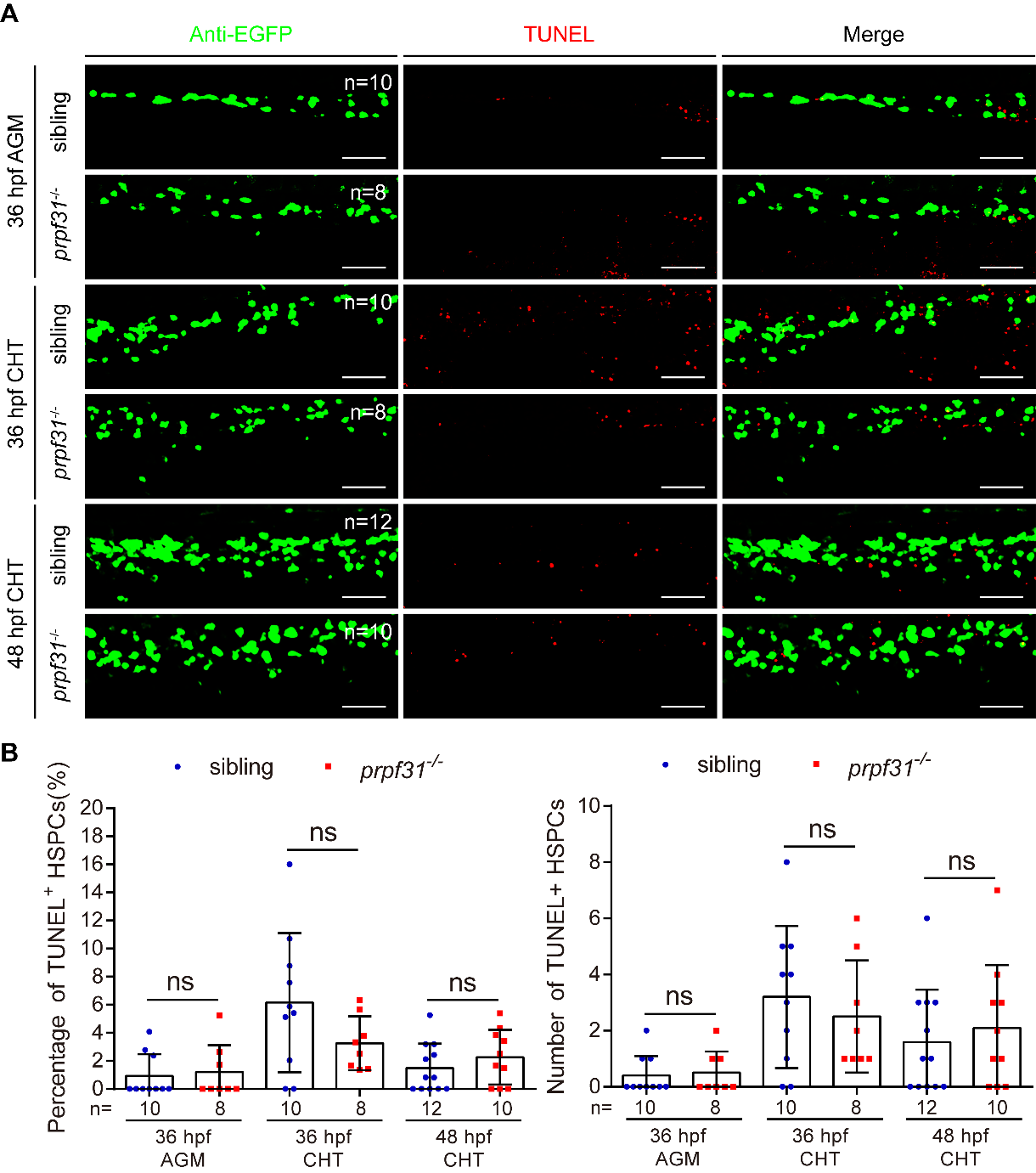


**Figure S5. HSPC deficiency in *prpf31*^-/-^ zebrafish is not mediated by the apoptosis of HSPCs.**

(**A**) Double staining of EGFP and TUNEL showed no significant increase of apoptotic HSPCs in the AGM and CHT of *prpf31*^-/-^ zebrafish at 36 and 48 hpf. The total number of embryos examined were indicated at the top right of each panel. Scale bars, 50 μm. (**B**) Quantification of the percentage and number of TUNEL^+^ HSPCs detected in (A). The total number of embryos examined were indicated below each column. Mean ± SD; unpaired two-tailed *t* test; ns, not significant, p > 0.05.


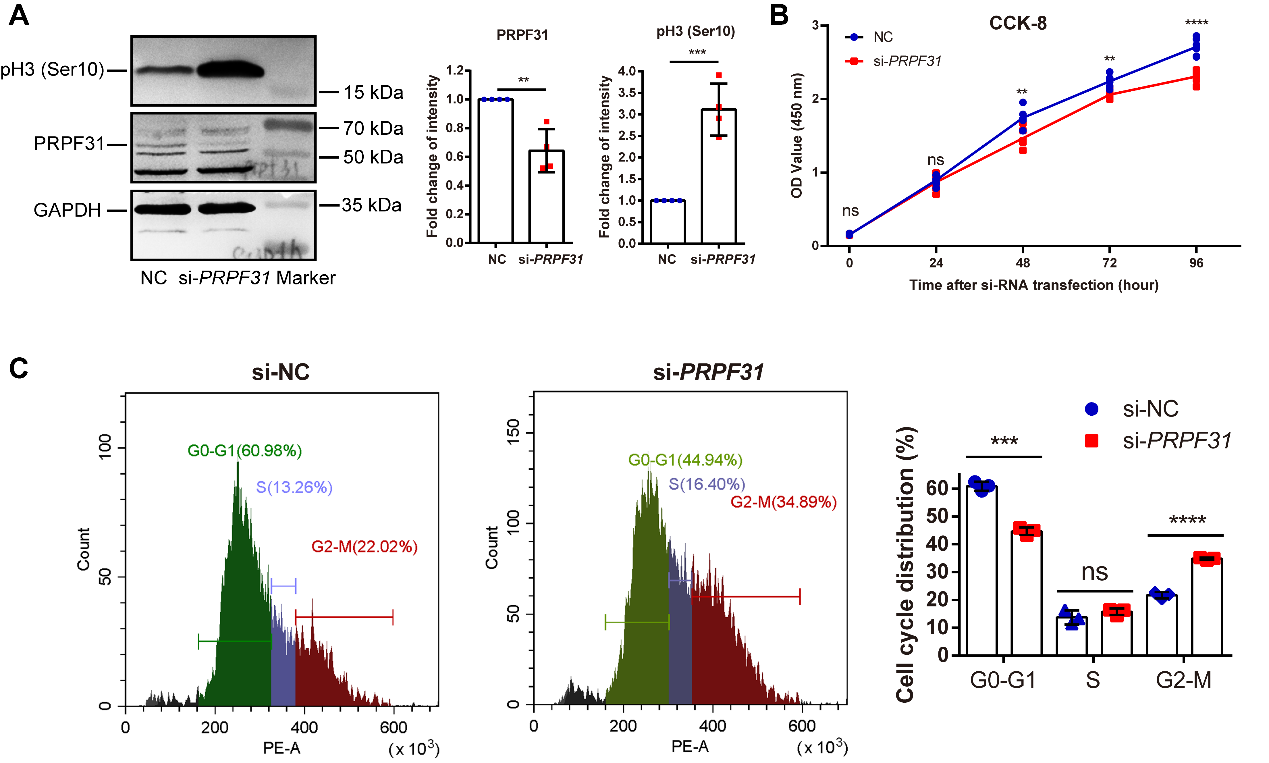


**Figure S6. Knockdown of *PRPF31* in HEK293 cell inhibited cell proliferation and caused M phase arrest.**

(**A**) Western blot analysis demonstrated reduced expression of PRPF31 protein and increased pH3 (Ser10) protein in si-*PRPF31* groups at 48 hours after si-RNA transfection. GAPDH was used as an internal control. Data were shown as mean ± SD of four independent experiments (n = 4); unpaired two-tailed *t* test; **p < 0.01, ***p < 0.001. (**B**) Cell Counting Kit-8 (CCK-8) assay indicated significantly reduced cell proliferation in si-*PRPF31* groups at 48, 72, and 96 hours after si-RNA transfection. Data were shown as mean ± SD of six independent experiments (n = 6); unpaired two-tailed *t* test; ns, not significant, **p < 0.01, ****p < 0.0001. (**C**) Flow cytometry analysis of the cell cycle showed increase in the percentage of cells in the G2/M phase arrest from 22.02% in NC HEK293 groups to 34.89% in si-*PRPF31* groups at 48 hours after si-RNA transfection. Data were shown as mean ± SD of three independent experiments (n = 3); unpaired two-tailed *t* test; ns, not significant, ***p < 0.001, ****p < 0.0001.


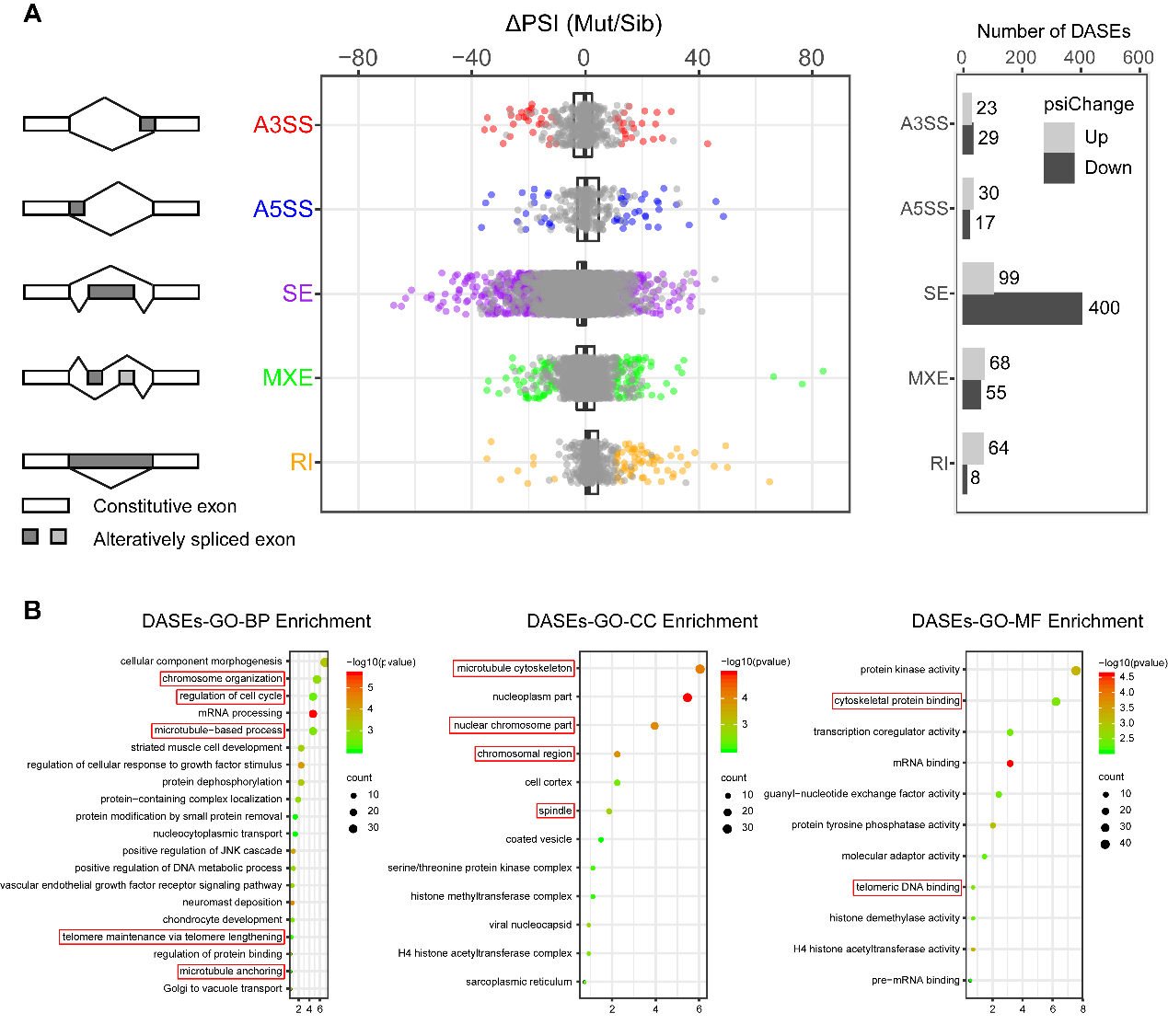


**Figure S7. Diagram showing differential alterative splicing event types and numbers, and functional gene ontology enrichment analysis showing enrichment in mitosis-related GO terms upon *prpf31* depletion.**

(**A**) Diagram of types and numbers of differential alterative splicing events. A3SS: alternative 3’ splice site; A5SS: alternative 5’ splice site; SE: skipped exon; MXE: mutually exclusive exons; RI: retained intron. (**B**) Functional GO enrichment analysis of differential alterative splicing genes corresponding to biological process (BP), cellular component (CC), and molecular function (MF) categories. Mitosis-related GO terms were represented (red boxes).

**Supporting datasets legends**

**Dataset S1. Information of DASEs of all splicing types.**

**Dataset S2. Information of functional GO enrichment analysis of differential alterative splicing genes corresponding to BP categories.**

**Dataset S3. Information of functional GO enrichment analysis of differential alterative splicing genes corresponding to CC categories.**

**Dataset S4. Information of functional GO enrichment analysis of differential alterative splicing genes corresponding to MF categories.**

**Dataset S5. Information of functional GO enrichment analysis of DASEs corresponding to three representative GOBP terms, i.e. chromosome organization (GO: 0051276), regulation of cell cycle (GO: 0051726), and microtubule-based process (GO: 0007017).**

**Dataset S6. Information on representative differential alternatively spliced mitosis-related genes confirmed by SqRT-PCR.**

**Dataset S7. All primers used in this article.**

**Supporting Video legends**

**Video S1. Live imaging of blood circulation in sibling embryos at 36 hpf.**

**Video S2. Live imaging of blood circulation in *prpf31*^-/-^ embryos at 36 hpf.**

**Video S3. Live imaging of blood circulation in sibling embryos at 3 dpf.**

**Video S4. Live imaging of blood circulation in *prpf31*^-/-^ embryos at 3 dpf.**
